# CMRegNet–An interspecies reference database for corynebacterial and mycobacterial regulatory networks

**DOI:** 10.1186/s12864-015-1631-0

**Published:** 2015-06-11

**Authors:** Vinicius A. C. Abreu, Sintia Almeida, Sandeep Tiwari, Syed Shah Hassan, Diego Mariano, Artur Silva, Jan Baumbach, Vasco Azevedo, Richard Röttger

**Affiliations:** Graduate Program in Bioinformatics, Institute of Biological Sciences, Federal University of Minas Gerais (Universidade Federal de Minas Gerais), Belo Horizonte, Minas Gerais Brazil; Institute of Biological Sciences, Federal University of Pará, Belém, Pará, Brazil; Department of Mathematics and Computer Science, University of Southern Denmark, Odense, Denmark; Computational Systems Biology, Max Planck Institute for Informatics, Campus E 2.1, 66123 Saarbrucken, Germany

## Abstract

**Background:**

Organisms utilize a multitude of mechanisms for responding to changing environmental conditions, maintaining their functional homeostasis and to overcome stress situations. One of the most important mechanisms is transcriptional gene regulation. In-depth study of the transcriptional gene regulatory network can lead to various practical applications, creating a greater understanding of how organisms control their cellular behavior.

**Description:**

In this work, we present a new database, CMRegNet for the gene regulatory networks of *Corynebacterium glutamicum* ATCC 13032 and *Mycobacterium tuberculosis* H37Rv. We furthermore transferred the known networks of these model organisms to 18 other non-model but phylogenetically close species (target organisms) of the CMNR group. In comparison to other network transfers, for the first time we utilized two model organisms resulting into a more diverse and complete network of the target organisms.

**Conclusion:**

CMRegNet provides easy access to a total of 3,103 known regulations in *C. glutamicum* ATCC 13032 and *M. tuberculosis* H37Rv and to 38,940 evolutionary conserved interactions for 18 non-model species of the CMNR group. This makes CMRegNet to date the most comprehensive database of regulatory interactions of CMNR bacteria. The content of CMRegNet is publicly available online via a web interface found at http://lgcm.icb.ufmg.br/cmregnet.

## Background

The ever growing number of completed genome sequencing projects has allowed for the extensive use of computational approaches for comparative genomics identifying potential transcriptional regulatory networks and key elements, such as transcription factor binding sites (TFBSs) [1]. These studies primarily focus on analyzing and describing regulatory elements that have been previously identified in model organisms and how this information may be applicable to organisms that have yet to be characterized. The comparative analysis of regulators combined with other genomic-context analysis techniques significantly improves the quality and accuracy of the functional gene annotations and the predictions of genes that may be involved in a variety of regulatory networks [2].

It is currently not possible, however, to decipher a complete regulatory network, even for a model organism. Potential reasons for this include an inability to simulate the different environmental conditions in which the organism lives in the laboratory and the inherent background noise of the existing wet-lab techniques. Even for the model organism *E. coli*, only a third of its transcriptional regulatory network (TRN) has been identified, even though a large number of studies with accurate data have been published on this organism [3]. The situation even worsens, when we focus on organisms like *Mycobacterium leprae*, which is the bacterium with the longest known duplication time and which does not grow in culture medium [4]. The aim of this study was to qualitatively and quantitatively contribute to the reconstruction of the transcriptional regulatory network between phylogenetically related species, specifically for species belonging to the CMNR group. The CMNR group belongs to the family of actinomycetes and consists of organisms belonging to the genera *Corynebacterium*, *Mycobacterium*, *Nocardia*, and *Rhodococcus*. Their phylogenetic correlation has been confirmed by 16S rDNA and rpoB DNA sequences analyses. The members of this group are Gram-positive bacteria that exhibit many peculiar features: (i) high G + C content, and (ii) a specific organization of the cell wall composed of mycolic acid, peptidoglycan and arabinolactano [5]. This group consists of several bacterial species that are of medical, veterinary, and biotechnological interest. Furthermore, some species of the CMNR group are important for industrial and biotechnological applications, such as *Corynebacterium glutamicum* and *Corynebacterium efficiens* [6]. In contrast, pathogenic species such as *Mycobacterium tuberculosis* and *Corynebacterium diphtheriae* (causing tuberculosis and diphtheria in humans, respectively) and *C. pseudotuberculosis*, which infects various animal species, especially small-size ruminants, are also prominent members of this group [7]. Because of their importance, several genomes of the CMNR group have been sequenced.

In the framework of this study, we aimed to computationally transfer the knowledge of the known TRN of the two-model organisms *Corynebacterium glutamicum* ATCC 1303 and *Mycobacterium tuberculosis* H37Rv to 18 other organisms of the CMNR group. The results were stored in an ontology-based database and are publicly available through the online platform CMRegNet. The platform also allows for several types of queries to access the database content and supports the reconstruction, analysis and visualization of the regulatory networks at different hierarchical levels.

CMRegNet is an interactive analysis platform for studying the transcriptional regulatory networks of the CMNR group of bacteria. The platform is publicly available at http://lgcm.icb.ufmg.br/cmregnet.

## Construction and content

### Overview of the CMRegNet system

The CMRegNet system is a database for transcriptional gene regulatory interactions of 20 (2 model organisms, 18 target organisms) different strains of the genera *Corynebacterium* and *Mycobacterium*. The system incorporates several bioinformatics data analysis procedures and information from different sources, in order to provide the user with all relevant information on regulatory interactions including the binding sites, protein sequences, gene annotations, and the genomic context of the regulation. The database itself runs on a MySQL 5.5 community server. The web service of CMRegNet is written in PHP (version 5.2.1) and delivered by an Apache web server (version 2.2.22).

As aforementioned in the introduction, the amount of known regulations is very scarce and only limited to a handful of model organisms. Thus, one key aspect of CMRegNet is the automated transfer of evolutionarily conserved regulations of these model organisms to the so called target organisms. For CMRegNet we exploit the same transfer pipeline which was already successfully used in CoryneRegNet [8–12] and MycoRegNet [13] in order to predict evolutionarily conserved regulations. CMRegNet may be regarded as the successor of the discontinued MycoRegNet [13] but was significantly extended: (1) we utilized ChiP-Seq data of *Mycobacterium tuberculosis* H37Rv in order to receive a comprehensive list of binding sites allowing *M. tuberculosis* to act as a model organism for the network transfer and (2) in contrast to comparable systems, CMRegNet bases the transfer of evolutionarily conserved regulations on two model organisms (*Corynebacterium* and *Mycobacterium*). This increases the predicted regulations in both, quantitative and qualitative aspects.

In order to transfer a regulation from a model organism to a target organism, we defined a simplified model of a gene regulation: A regulation requires three main drivers, namely the transcription factor, the target gene and the corresponding binding site in the upstream region of the target gene. We consider a regulation as evolutionarily conserved, if in the target organism (1) the transcription factor is conserved, (2) the target gene is conserved and (3) the target gene possesses the binding site for the transcription factor in its upstream region.

In order to detect conserved genes (i.e., homologous genes), we decided to perform a homology detection based on the clustering of the protein sequences, i.e., the reported clusters form the groups of homologous proteins. Generally, for the homology detection with a clustering tool, a similarity measure between the proteins and a meaningful parameter setting for the employed clustering tool are required. For CMRegNet we use transitivity clustering (TransClust) and followed the approach described in [14] which suggest the usage of a BLAST all-vs.-all run on the protein sequences with an E-value cut-off of 10 as similarity function. The threshold (the parameter of TransClust) was selected following the suggestions in [15]. In this study, the authors developed a measure for judging the quality of a clustering for homology detection by basically evaluating two aspects of the cluster-size distribution: (1) the number of genes in the core-genome (genes shared by all organisms) and (2) the number of unrealistically large clusters (which most likely contain false positives). The idea is now to find that threshold which maximizes (1) while minimizing (2). In the original study, the authors suggest to pick a threshold between 34 and 61 for mycobacteria and 27 and 53 for corynebacteria. For CMRegNet, we decided to use a rather non-stringent threshold of 30 which is in the middle of the two lower bounds of both suggested threshold ranges. We decided to do so because we (1) have proteins from both genera, *Corynebacterium* and *Mycobacterium* and (2) the homology detection is only one of three criteria (as described above) for the prediction of an evolutionarily conserved regulation. Thus we are convinced that this selection of the threshold does not increase the false-positive rate while providing a large basis of potential homologous proteins for the regulation transfer.

We used PoSSuMsearch [16] with a p-value cut-off of 10 in order to identify possible binding sites in the upstream region (-540 pb … +40 pb relative to start codon) of the potential target gene. With that information, we can identify evolutionarily conserved regulations in the target organisms.

The transferred regulations undergo an additional refinement process utilizing operon predictions obtained from MicrobesOnline [17]. A regulation is only considered conserved, if the target gene is also the first gene in an operon. If this condition holds, all genes in the operon of the target organism are consequentially predicted to be regulated by the transcription factor in question.

In the case that the same regulation is predicted by the network transfer of both model organisms, we store and display two *in silicio* evidences for this regulation and refer to the two experimental validated regulations in the model organisms.

To sum up, for the target organisms, we require gene annotations and the operon predictions. For the model organisms we additionally need information of the regulatory interactions including the binding sites of the involved transcription factors. In the following, we describe all utilized data sources for CMRegNet.

### Target organisms

For the 18 target organisms included into CMRegNet, the publicly available sequences and annotation data from the National Center for Biotechnology Information (NCBI) were retrieved [18]. The operon prediction data was provided by an integrated portal for comparative and functional genomics, MicrobesOnline [17].

### Model organisms

For both model organisms, we obtained the operon predictions as well as the gene annotations from the same sources as for the target organisms. For the model organisms, additional information on the regulatory interactions had to be derived. The reconstruction of both regulatory networks is mainly composed of experimental data derived from the literature. In the following section, we describe the additional data sources used.

### *Corynebacterium glutamicum* ATCC 13032

With CoryneRegNet [12], there already exists a reference database and analysis platform for corynebacterial gene regulatory networks. The biological content of CoryneRegNet comprehensively covers transcriptional regulations in the model organism *C. glutamicum* ATCC 13032 and provides all necessary information for CMRegNet, inclue TFBS and regulation. We extracted a total of 1,441 known regulatory interactions, 520 TFBS, 97 regulators, and their respective target genes. The data of CoryneRegNet is derived from various wet-lab experiments such as ChiP-ChiP, ChiP-Seq, and microarrays, but mostly derived from microarray experiments [8].

### *Mycobacterium tuberculosis* H37Rv

For *M. tuberculosis* H37Rv, despite being a well-established model organism, no such database providing necessary support for transcriptional gene regulatory networks exists. However, for *M. tuberculosis* H37Rv, the Tuberculosis Database (TBDB) serves as a database collecting all tuberculosis related research resources, e.g., expression data, metabolomic networks, relevant publications, and many more. Especially, TBDB hosts several omics data from multiple strains of *M. tuberculosis*, as well as data related to the genera *Mycobacterium* [16, 19]. In contrast to *C. glutamicum*, we do not have the TFBS information of each mapped regulator for the genome of *M. tuberculosis* H37Rv. However, TBDB provides for every regulator the upstream region of the target genes which most likely contain the TFBS. In order to extract the actual binding sites required for CMRegNet, we performed the following strategy.

### Retrieving the binding sites for *M. tuberculosis*

**Fig. 1:**
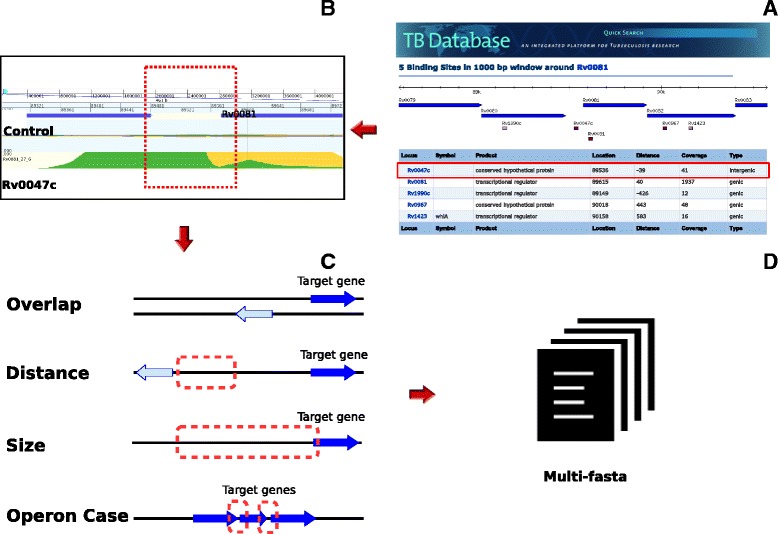
Binding sites pipeline. **(a)** Table from TBDB with relation (gene target – regulator) and their respective coordinates; **(b)** GenomeView to visualize in the genomic context; **(c)** The different cases of inconsistency: (i) Overlap - When it has the overlapping region with neighbour’s intragenic regions; (ii) Distance - When the region is more distant than the chosen threshold; (iii) Size - When the size transcends the threshold; (iv) Operon - case where peak regions are intraoperon; and **(d)** Output file, each multi-FASTA corresponds a regulator

Through its “Search Regulatory Binding Sites” option, the TBDB provides a table of possible regulatory genes for a given gene of interest (Fig. 1a). The information is based on ChIP-Seq experiments. We processed the following core information: (1) gene regulator, (2) the distance of start codon of the target gene, and (3) the start and stop coordinates of a region of possible TFBS.

However, to predict TFBS in these regions, we found some inconsistencies, such as overlap, distance, size and peaks, within the operons (Fig. 1a and 1c). Although, there are some reported cases where the TFBS is found in regions with a high overlap and more significant distances in *M. tuberculosis* [20], we followed a more stringent criterion to reduce the number of false positives. We limited the peak regions to an area between +40 bps to -540 pbs in relation to the target gene. A Perl script was used to filter the data obtained by TBDB. For each regulator a FASTA file consisting of all sequences possibly containing the TFBS was created (Fig. 1d). These FASTA files formed the input for a subsequent TFBS prediction using MEME-ChIP [21].

**Fig. 2:**
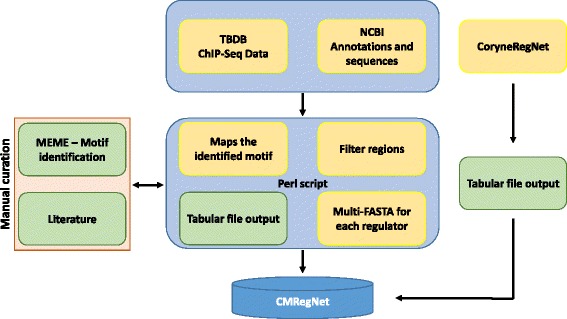
Schematic overview illustrating the retrieval of binding sites information of both model organisms *M. tuberculosis* H37Rv and *C. glutamicum* ATCC 13032 using TBDB and CoryneRegNet as main sources

MEME-Chip is a tool used for predicting large-scale motif sequences. We performed a MEME run on each FASTA file using the default parameters. An extensive literature search was performed to look for experimental data on TFBS. Whenever experimental evidence for a TFBS was available, we utilized this additional information by becoming more stringent in the setting of the “Maximum width motif” parameter according to the motif reported in the literature. An overview of the pipeline analyses is depicted in Fig. 2.

At this point, we have acquired all required data of the model organisms, namely the set of regulators and target genes with their corresponding TFBS. With this information we are able to run the previously described network transfer pipeline and transfer all evolutionarily conserved regulation from both model organisms to all 18 target organisms.

## Utility and Discussion

The utilization of experimental data of two phylogenetically close model organisms (*C. glutamicum and M. tuberculosis*) combined with the comparative genomics approach for transferring the gene regulatory network makes the CMRegNet a reference database for studying regulatory networks of organism in the CMNR group. Table 1 summarizes the database content of CMRegNet. The CMRegNet is divided into two evidence levels: (1) experimental and (2) predicted. The experimental version only contains experimentally validated regulations whereas the predicted version additionally contains the transferred evolutionarily conserved regulations as well. In Table 1, we depict all regulations stored in the predicted version of CMRegNet. Whenever a regulation was predicted as conserved based on both model organisms, it was counted as two regulations in the table.

**Table 1:** This table summarizes the database content of CMRegNet

Organism	Genes	Reg	TG	TFBS	CG	MT	Both	NRs
*C. aurimucosum* ATCC 700975	2531	31	448	420	294	264	108	666
*C. diphtheriae* NCTC 13129	2272	37	379	470	270	68	210	548
*C. efficiens* YS-314	2950	44	601	537	443	262	248	953
*C. glutamicum* ATCC 13032	3058	94	723	452	1314	0	0	1314
*C. glutamicum* R	3052	51	724	773	638	282	312	1232
*C. jeikeium* K411	2104	25	418	440	212	156	223	591
*C. kroppenstedtii* DSM 44385	2018	24	399	321	199	114	279	591
*C. pseudotuberculosis* 1002	2057	31	408	339	285	91	239	615
*C. pseudotuberculosis* C231	2053	29	409	338	281	93	234	608
*C. pseudotuberculosis* FRC41	2110	30	437	355	303	93	250	646
*C. urealyticum* DSM 7109	2024	25	377	356	176	132	262	570
*M. abscessus*	4920	45	1374	1855	198	2156	541	2895
*M. avium* 104	5120	40	1489	2358	101	2676	467	3244
*M. bovis* BCG str. Pasteur 1173P2	3948	53	2195	4117	115	5209	407	5731
*M. leprae* Br4923	1604	17	450	335	107	326	208	641
*M. marinum* M	5423	44	1919	3805	121	4190	742	5053
*M. smegmatis* str. MC2 155	6717	50	1555	2299	266	2366	614	3246
*M. tuberculosis* F11	3941	52	1934	3988	106	4852	294	5252
*M. tuberculosis* H37Ra	4034	54	2380	4391	142	5654	498	6294
*M. tuberculosis* H37Rv	4003	40	1458	2466	0	2466	0	2466

Legend: Genes = total number of genes; Reg = total number of regulatory genes; TG = number of target genes; TFBS = total number of binding sites; CG = number of regulations exclusively transferred from *Corynebacterium glutamicum* ATCC 13032; MT = number of regulations exclusively transferred from *Mycobacterium tuberculosis* H37Rv; Both = regulations predicted by both model organisms; NRs = total number of regulations

So far, databases similar to CMRegNet were limited to only one model organism, e.g., in [12, 22]. Considering the scarce knowledge we have on regulatory interaction even for heavily studied model organisms [3], the restriction to one model organism poses one of the most prominent limitations of the automated network transfer [23]. Apparently, a regulation can only be transferred to a target organism, when it was experimentally validated in model organisms in the first place. Here, for the first time we utilized two model organisms which are phylogenetically close but with different life-styles. This allows us to overcome the limitations imposed by the use of a single model. We illustrate the potential of CMRegNet for *Mycobacterium leprae*, an etiologic agent of leprosy disease. Note, that *M. leprae* has a atypical *g*enome within the CMNR group: a large number of pseudogenes, accumulation of insertion sequence, lowered G + C content, which all are hallmarks of reductive evolution, and may reflect passage through an evolutionary bottleneck [24]. Nevertheless, CMRegNet was able to transfer 641 conserved interactions in total, 107 of them from *C. glutamicum*, 326 from *M. tuberculosis,* and 208 were conserved in both models. This is a strong indicator for the power and utility of using two model organisms instead of only one.

All large-scale integrative databases, such as CMRegNet, are facing the same challenge of integrating data from various sources derived by different techniques. The literature data from *C. glutamicum* ATCC 13032 shows a great diversity of techniques applied to study gene regulations. Exemplarily, the characterization of the global gene regulator glxR varies from ChIP-Seq techniques [25] over various studies of the gene transcription [26–29] to analyses derived from microarray and PCR experiments.

In contrast to *C. glutamicum* ATCC 13032, the data from *M. tuberculosis* H37Rv utilized in CMRegNet originates mostly from the “Flag-tagged” ChIP-Seq data. This approach allows studying a large number of transcription factors, without the necessity of previous knowledge of the conditions that normally induce its expression, and the identification of regions enriched in SLFT [20]. This enabled the usage of *M. tuberculosis* as a model organism in the first place.

The difference in the data sources between both model organisms is consequentially also reflected in the number of detected regulations (compare Table 1): 94 regulators responsible for a total of 1314 regulations are found in *C. glutamicum* whereas 40 regulators in *M. tuberculosis* are responsible for 2466 regulations.

In order to reflect this diversity of evidence in the provided data, every regulation stored in CMRegNet is linked to the source of its evidence so that researchers are able to make an informed decision whether a certain regulation is reliable enough or not for their purpose.

Furthermore, the automated transfer of evolutionarily conserved regulations has a main limitation: Only regulations already known in model organisms can possibly transferred to the target organisms. Additionally, even for these highly studied model organisms, the currently known regulatory network is far from complete [3]. Until this work, the databases similar to CMRegNet were limited only to one model organism [8–12, 30–32] which was extended to a second model organism for CMRegNet; but nevertheless the amount of information of the target organisms is strictly limited by the available information on the model organisms.

### Navigation

CMRegNet is accessible by a user-friendly online interface. As already mentioned, CMRegNet provides the user with two different choices of evidence level: (I) experimental, which concentrates on the dataset with experimental verification, and (II) predicted, which additionally provides the transferred regulations. After selecting the evidence level, the user is presented an overview page consisting of a list of organisms, summary statistics, and search box for almost arbitrary queries.

In the following, we demonstrate the power of CMRegNet using the gene Rv0081 as an example. The Rv0081 gene of *M. tuberculosis* H37Rv encodes a transcriptional regulator HTH-type, a member of the dormancy regulon. The gene was first extensively described by Black et al. [33]. Rv0081 is of biotechnological importance and serves as an immunogenic antigen, inducing interferon-gamma, indicating that this might be a good vaccine candidate. It is hoped that this regulon will give insight into the latent or dormant phase of infection [33].

**Fig 3:**
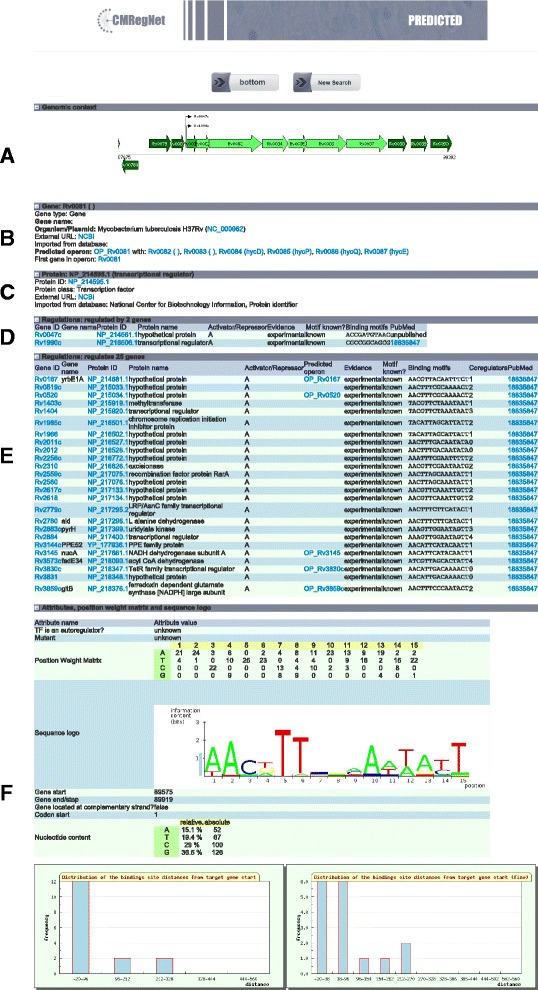
The expandable list summarizes the relevant data for the reconstruction of regulatory networks of the gene Rv0081 of *M. tuberculosis.* **(a)** The operon membership of the selected gene; **(b)** gene/protein information, providing links to the genome annotation deposited at the NCBI; **(c/d/e)** regulations, summarizing all available information on the gene regulatory network of the selected gene; **(f)** attributes, providing PSSMs and sequence logos of the predicted Rv0081 binding sites consensus. The height of each letter within an individual stack represents the nucleotide’s frequency relative to the particular motif position; thus, the stack according to the respective position indicates the degree of a nucleotide’s conservation

After identifying the genome and the gene of interest, the user receives all relevant information of the gene and its embedding in the regulatory network (Fig. 3). The results are displayed in an expandable list. In these sections, users can retrieve the following information: (a) the context of the gene in regard to the genome, (b) gene information, (c) protein information, (d) regulating genes and in case of a transcription factor additionally (e) a list of its target genes and (f) information about the corresponding binding site. Furthermore, the user can search in the upstream region of the gene of interest for potential binding sites and–in case of transcription factors–search in the upstream region of all other genes for its associated binding site. In Fig. 3, it can also be observed that the genes that are part of the *Rv0081* operon are highlighted in “light green”. The CMRegNet allows clicking on any of the gene, which immediately redirect to another page with details of another gene.

**Fig. 4:**
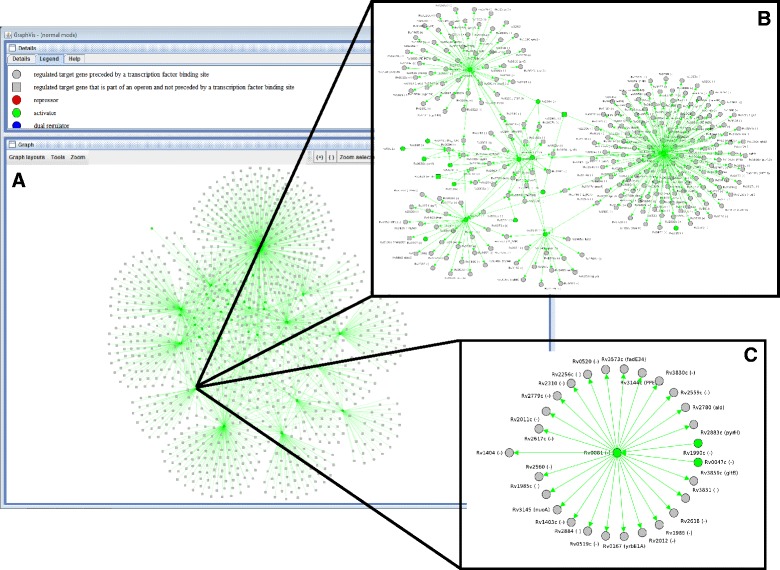
Reconstruction of the gene network of Rv0081 of *M. tuberculosis*. The figure shows different levels of regulation. **(a)**, **(b)**, and **(c)** were created using a cut-off of 3, 2 and 1 respectively. The layout of **(c)** is circular, of **(a)** and **(b)** organic. The computed results showed that Rv0081 regulated 25 genes and a single regulator was identified for this gene, using a cut-off 1 **(c)**

CMRegNet also provides visualization of the network using GraphVis, which is a Java applet. The user can either display the whole regulatory network of the organism, or only the relevant part for a certain gene of interest. GraphVis allows the user to zoom into the chart, apply different layout styles, remove, add and edit selected elements (e.g., researchers can add new regulations of their current study in the visualization) or obtain detailed information about the selected genes. Figure 4 depicts the regulatory network of Rv0081.

**Fig. 5:**
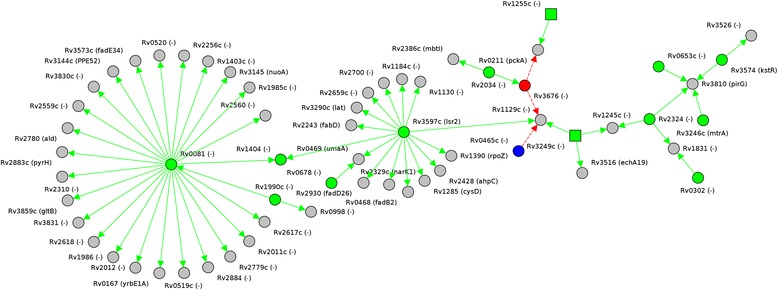
Screenshot of GraphVis after the manual integration of eleven additional genes

Furthermore, GraphVis allows for the projection of expression levels onto the currently loaded network. This data can either be chosen from the integrated stimulon database or manually added. In Fig. 5, we manually added the gene expression results presented in [22]. In this work, Fontán et al. observed that after infecting IFN-γ-treated BMM-differentiated THP-1 cells, 11 genes were also up regulated, namely: Rv0080, Rv0081, Rv2028c, *pfkB*, Rv2030c, *acr*, *acg*, Rv2626c, Rv3133c, *fadE24*, and *fadE23.* Such information can easily be integrated and visualized by GraphVis.

As a second example demonstrating the strength of CMRegNet, we present the findings of the gene *glxR*. This gene is a DNA-binding transcriptional regulator of the FNR-CRP protein family and is well studied in genus *Corynebacterium* [26, 28]. However, this gene is only little studied in the genus *Mycobacterium*, with no available information on regulations of this gene so far. For example, in *M. abscessus* the gene MAB_0416c was identified as a homologous protein to *glxR* and through the network transfer, CMRegNet now shows 102 conserved regulations.

The analysis of the Rv0081 and *glxR* gene demonstrated the in-depth analysis capabilities of CMRegNet for gene regulatory networks.

## Conclusion

In this work, we have reconstructed the regulatory network of two important pathogen models of the genera Corynebacterium and Mycobacterium, including publicly available experimentally validated data. This data has been computationally transferred to 18 related organisms (see Table 1), making it so far the largest database of regulatory network targeted to the CMNR group. CMRegNet provides the data in an easily accessibly manner allowing for efficient analyzes of the regulatory networks and furthermore provides the user with the possibility to integrate own data into the analysis. These features support researchers in designing their future wet-lab experiments.

Furthermore, we constantly screen the relevant literature and regularly extend the database with newly published regulations.

## Availability and requirements

The CMRegNet database is freely available for non-commercial use at http://lgcm.icb.ufmg.br/cmregnet. The GraphVis applet requires a browser with a Java plug-in installed.
